# Supplementary material for: Regional response to light illuminance across the human hypothalamus
Source: eLife. 2024 Oct 28;13:RP96576. doi: 10.7554/eLife.96576 (PMC11517251; doi:10.7554/eLife.96576)
Supplement: Supplementary file 1. — (a) Demographics of study sample. (b) Light characteristics. (c) Post hoc contrasts between illuminances within each hypothalamus subpart during the executive task. (d) Post hoc contrasts between illuminances within each hypothalamus subpart during the emotional task. (e) Post hoc contrasts between hypothalamus subpart for each illuminance during the executive task. (f) Post hoc contrasts between hypothalamus subpart for each illuminance during the emotional task. (g) Association between performance to the 2-back task and the activity of each hypothalamus subpart during each illuminance. [file elife-96576-supp1.docx]

**ONLINE SUPPLEMENTARY INFORMATION**

**Regional response to illuminance across the human hypothalamus**

Islay Campbell^1#^, Roya Sharifpour^1#^, Jose Fermin Balda Aizpurua^1^, Elise Beckers^1,2^, Ilenia Paparella^1^, Alexandre Berger^1,3,4^, Ekaterina Koshmanova^1^, Nasrin Mortazavi^1^, John Read^1^, Mikhail Zubkov^1^, Puneet Talwar^1^, Fabienne Collette^1^, Siya Sherif^1^, Christophe Phillips^1^, Laurent Lamalle^1^, Gilles Vandewalle^1^

^1^GIGA-Cyclotron Research Centre-In Vivo Imaging, University of Liège, Liège, 4000 Belgium

^2^Faculty of Health, Medicine and Life Sciences, School for Mental Health and Neuroscience, Alzheimer Centre Limburg, Maastricht University, Maastricht, 6229 ER The Netherlands.

^3^Institute of Neuroscience (IoNS), Department of Clinical Neuroscience,Université Catholique de Louvain (UCLouvain), Woluwe-Saint-Lambert, 1200 Belgium.

^4^Synergia Medical SA, Mont-Saint-Guibert, 1435 Belgium.

^#^ shared first authorship

*Corresponding author: Gilles Vandewalle, GIGA-Cyclotron Research Centre-In Vivo Imaging, Bâtiment B30, 8 Allée du Six Août, University of Liège-Sart Tilman, 4000 Liège, Belgium.

**Supplementary Table S1a. Demographics of study sample.**

|  | **Total Sample** | **Executive Task** | **Emotional Task** |
| --- | --- | --- | --- |
| **Number of Participants** | 30 | 26 | 26 |
| **Age** | 24.2 ± 2.9 | 24.3 ± 3.0 | 24.4 ± 3.0 |
| **Sex (M)** | 11 | 10 | 10 |
| **Mood (BDI-II)** | 7.5 ± 7.0 | 6.7 ± 6.0 | 8.0 ± 7.3 |
| **Anxiety (BAI)** | 5.0 ± 4.1 | 4.8 ± 3.8 | 5.1 ± 4.3 |
| **Sleep quality (PSQI)** | 4.0 ± 2.6 | 3.7 ± 2.5 | 4.0 ± 2.7 |
| **Seasonality (SPAQ)** | 1.1 ± 0.8 | 1.2 ± 0.8 | 1.2 ± 0.8 |
| **Chronotype (HO)** | 48.7 ± 8.0 | 48.9 ± 8.2 | 48.7 ± 7.8 |
| **Daytime sleepiness (ESS)** | 6.5 ± 3.0 | 6.3 ± 3.0 | 6.2 ± 3.0 |
| **Years of Education** | 14.5 ± 3.1 | 14.5 ± 3.2 | 14.2 ± 3.2 |
| **Sleep duration (night before fMRI protocol – sleep diary based)** | 7.9 ± 0.7 | 7.8 ± 0.7 | 7.9 ± 0.7 |

Total number of participants who completed the study, and the number of participants included for each task (some participants had missing/corrupted data, see methods). BDI-II, Beck's Depression Inventory; BAI, Beck Anxiety Inventory; PSQI, Pittsburgh Sleep Quality Index; SPAQ, Seasonal Pattern Assessment Questionnaire; HO, Horne and Östberg; ESS, Epworth Sleepiness Scale. Refer to the method for the references to the scales and questionnaires.

**Supplementary Table S1b. Light characteristics.**

|  | **Low BEL** | **Mid BEL** | **High BEL** | **Orange** |
| --- | --- | --- | --- | --- |
| **Lux** | 47 | 116 | 240 | 7.5 |
| **Peak Spectral Irradiance (nm)** | 460 | 460 | 460 | 590 |
| **Melanopic EDI (lux; ipRGCs)** | 37 | 92 | 190 | 0.16 |
| **Rhodopic EDI (lux; Rods)** | 39 | 97 | 201 | 0.94 |
| **Cyanopic EDI (lux; S-cones)** | 32 | 79 | 163 | 0 |
| **Chloropic EDI (lux; M-cones)** | 44 | 110 | 227 | 5 |
| **Erythropic EDI (lux ; L-cones)** | 46 | 113 | 233 | 8 |
| **Irradiance (µW/cm²)** | 15 | 36 | 75 | 1.4 |
| **Photon flux(1/cm²/s)** | 4.12E+13 | 1.02E+14 | 2.10E+14 | 4.24E+12 |
| **Log Photon Flux (log₁₀ (1/cm²/s)** | 13.61 | 14.01 | 14.32 | 12.63 |
| **Narrowband peak** | - | - | - | 589 |
| **Narrowband FWHM** | - | - | - | 10 |

Detailed characteristics of the four conditions used in fMRI protocol. Blue enriched (BEL) (low, mid, and high) and monochromatic (589nm). ipRGCs: intrinsically photosensitive retinal ganglion cells. FWHM: full width at half maximum.

**Supplementary Table S1c. Post hoc contrasts between illuminances within each hypothalamus subpart during the executive task**

| **Hypothalamus subpart** | **illuminance** | **vs. illuminance** | **t-value** | **p-value** |
| --- | --- | --- | --- | --- |
| **1 (inferior-anterior)** | 0 | 0.16 | 2.43 | **0.0151** |
| **1 (inferior-anterior)** | 0 | 37 | 2.59 | **0.0098** |
| **1 (inferior-anterior)** | 0 | 92 | 1.65 | 0.0993 |
| **1 (inferior-anterior)** | 0 | 190 | 3.30 | **0.0010** |
| **1 (inferior-anterior)** | 0.16 | 37 | 0.17 | 0.8683 |
| **1 (inferior-anterior)** | 0.16 | 92 | -0.78 | 0.4330 |
| **1 (inferior-anterior)** | 0.16 | 190 | 0.86 | 0.3886 |
| **1 (inferior-anterior)** | 37 | 92 | -0.95 | 0.3445 |
| **1 (inferior-anterior)** | 37 | 190 | 0.69 | 0.4892 |
| **1 (inferior-anterior)** | 92 | 190 | 1.65 | 0.0999 |
| **2 (superior-anterior)** | 0 | 0.16 | 0.79 | 0.4313 |
| **2 (superior-anterior)** | 0 | 37 | 0.76 | 0.4480 |
| **2 (superior-anterior)** | 0 | 92 | 1.37 | 0.1722 |
| **2 (superior-anterior)** | 0 | 190 | 1.15 | 0.2520 |
| **2 (superior-anterior)** | 0.16 | 37 | -0.02 | 0.9810 |
| **2 (superior-anterior)** | 0.16 | 92 | 0.58 | 0.5628 |
| **2 (superior-anterior)** | 0.16 | 190 | 0.36 | 0.7198 |
| **2 (superior-anterior)** | 37 | 92 | 0.60 | 0.5490 |
| **2 (superior-anterior)** | 37 | 190 | 0.38 | 0.7036 |
| **2 (superior-anterior)** | 92 | 190 | -0.22 | 0.8259 |
| **3 (posterior)** | 0 | 0.16 | -1.32 | 0.1873 |
| **3 (posterior)** | 0 | 37 | -1.22 | 0.2240 |
| **3 (posterior)** | 0 | 92 | -2.14 | **0.0323** |
| **3 (posterior)** | 0 | 190 | -2.35 | **0.0190** |
| **3 (posterior)** | 0.16 | 37 | 0.10 | 0.9180 |
| **3 (posterior)** | 0.16 | 92 | -0.82 | 0.4101 |
| **3 (posterior)** | 0.16 | 190 | -1.03 | 0.3037 |
| **3 (posterior)** | 37 | 92 | -0.93 | 0.3542 |
| **3 (posterior)** | 37 | 190 | -1.13 | 0.2578 |
| **3 (posterior)** | 92 | 190 | -0.21 | 0.8375 |
| **4 (inferior-tubular)** | 0 | 0.16 | 2.15 | **0.0316** |
| **4 (inferior-tubular)** | 0 | 37 | 2.21 | **0.0271** |
| **4 (inferior-tubular)** | 0 | 92 | 2.80 | **0.0052** |
| **4 (inferior-tubular)** | 0 | 190 | 3.27 | **0.0011** |
| **4 (inferior-tubular)** | 0.16 | 37 | 0.06 | 0.9518 |
| **4 (inferior-tubular)** | 0.16 | 92 | 0.65 | 0.5176 |
| **4 (inferior-tubular)** | 0.16 | 190 | 1.12 | 0.2624 |
| **4 (inferior-tubular)** | 37 | 92 | 0.59 | 0.5575 |
| **4 (inferior-tubular)** | 37 | 190 | 1.06 | 0.2891 |
| **4 (inferior-tubular)** | 92 | 190 | 0.47 | 0.6356 |
| **5 (superior-tubular)** | 0 | 0.16 | 0.01 | 0.9882 |
| **5 (superior-tubular)** | 0 | 37 | 0.84 | 0.3986 |
| **5 (superior-tubular)** | 0 | 92 | 0.86 | 0.3920 |
| **5 (superior-tubular)** | 0 | 190 | 0.58 | 0.5604 |
| **5 (superior-tubular)** | 0.16 | 37 | 0.83 | 0.4069 |
| **5 (superior-tubular)** | 0.16 | 92 | 0.84 | 0.4002 |
| **5 (superior-tubular)** | 0.16 | 190 | 0.57 | 0.5704 |
| **5 (superior-tubular)** | 37 | 92 | 0.01 | 0.9905 |
| **5 (superior-tubular)** | 37 | 190 | -0.26 | 0.7934 |
| **5 (superior-tubular)** | 92 | 190 | -0.27 | 0.7842 |

**Supplementary Table S1d. Post hoc contrasts between illuminances within each hypothalamus subpart during the emotional task**

| **Hypothalamus subpart** | **Illuminance** | **Vs. illuminance** | **t-value** | **p-value** |
| --- | --- | --- | --- | --- |
| **1 (inferior-anterior)** | 0 | 0.16 | -1.19 | 0.2324 |
| **1 (inferior-anterior)** | 0 | 37 | 1.29 | 0.1979 |
| **1 (inferior-anterior)** | 0 | 92 | 2.03 | **0.0431** |
| **1 (inferior-anterior)** | 0 | 190 | 2.25 | **0.0248** |
| **1 (inferior-anterior)** | 0.16 | 37 | 2.48 | **0.0132** |
| **1 (inferior-anterior)** | 0.16 | 92 | 3.22 | **0.0013** |
| **1 (inferior-anterior)** | 0.16 | 190 | 3.44 | **0.0006** |
| **1 (inferior-anterior)** | 37 | 92 | 0.74 | 0.4616 |
| **1 (inferior-anterior)** | 37 | 190 | 0.96 | 0.3379 |
| **1 (inferior-anterior)** | 92 | 190 | 0.22 | 0.8243 |
| **2 (superior-anterior)** | 0 | 0.16 | -0.14 | 0.8910 |
| **2 (superior-anterior)** | 0 | 37 | 1.14 | 0.2539 |
| **2 (superior-anterior)** | 0 | 92 | 2.86 | **0.0043** |
| **2 (superior-anterior)** | 0 | 190 | 3.49 | **0.0005** |
| **2 (superior-anterior)** | 0.16 | 37 | 1.28 | 0.2013 |
| **2 (superior-anterior)** | 0.16 | 92 | 3.00 | **0.0028** |
| **2 (superior-anterior)** | 0.16 | 190 | 3.63 | **0.0003** |
| **2 (superior-anterior)** | 37 | 92 | 1.72 | 0.0853 |
| **2 (superior-anterior)** | 37 | 190 | 2.35 | **0.0190** |
| **2 (superior-anterior)** | 92 | 190 | 0.63 | 0.5310 |
| **3 (posterior)** | 0 | 0.16 | -1.24 | 0.2151 |
| **3 (posterior)** | 0 | 37 | 0.13 | 0.8954 |
| **3 (posterior)** | 0 | 92 | -0.15 | 0.8799 |
| **3 (posterior)** | 0 | 190 | -2.17 | **0.0299** |
| **3 (posterior)** | 0.16 | 37 | 1.37 | 0.1704 |
| **3 (posterior)** | 0.16 | 92 | 1.09 | 0.2763 |
| **3 (posterior)** | 0.16 | 190 | -0.93 | 0.3506 |
| **3 (posterior)** | 37 | 92 | -0.28 | 0.7775 |
| **3 (posterior)** | 37 | 190 | -2.31 | **0.0213** |
| **3 (posterior)** | 92 | 190 | -2.02 | **0.0433** |
| **4 (inferior-tubular)** | 0 | 0.16 | 0.06 | 0.9486 |
| **4 (inferior-tubular)** | 0 | 37 | 1.01 | 0.3134 |
| **4 (inferior-tubular)** | 0 | 92 | 2.54 | **0.0113** |
| **4 (inferior-tubular)** | 0 | 190 | 2.42 | **0.0155** |
| **4 (inferior-tubular)** | 0.16 | 37 | 0.94 | 0.3454 |
| **4 (inferior-tubular)** | 0.16 | 92 | 2.47 | **0.0135** |
| **4 (inferior-tubular)** | 0.16 | 190 | 2.36 | **0.0185** |
| **4 (inferior-tubular)** | 37 | 92 | 1.53 | 0.1262 |
| **4 (inferior-tubular)** | 37 | 190 | 1.42 | 0.1571 |
| **4 (inferior-tubular)** | 92 | 190 | -0.11 | 0.9087 |
| **5 (superior-tubular)** | 0 | 0.16 | 0.04 | 0.9679 |
| **5 (superior-tubular)** | 0 | 37 | 1.85 | 0.0651 |
| **5 (superior-tubular)** | 0 | 92 | 1.71 | 0.0870 |
| **5 (superior-tubular)** | 0 | 190 | 1.10 | 0.2713 |
| **5 (superior-tubular)** | 0.16 | 37 | 1.81 | 0.0711 |
| **5 (superior-tubular)** | 0.16 | 92 | 1.67 | 0.0946 |
| **5 (superior-tubular)** | 0.16 | 190 | 1.06 | 0.2892 |
| **5 (superior-tubular)** | 37 | 92 | -0.13 | 0.8939 |
| **5 (superior-tubular)** | 37 | 190 | -0.75 | 0.4558 |
| **5 (superior-tubular)** | 92 | 190 | -0.61 | 0.5403 |

**Supplementary Table S1e. Post hoc contrasts between hypothalamus subpart for each illuminance during the executive task**

| **Illuminance** | **subpart** | **vs. subpart** | **t-value** | **p-value** |
| --- | --- | --- | --- | --- |
| **0** | 1 (inferior-anterior) | 2 (superior-anterior) | 1.25 | 0.2106 |
| **0** | 1 (inferior-anterior) | 3 (posterior) | 1.95 | 0.0511 |
| **0** | 1 (inferior-anterior) | 4 (inferior-tubular) | 0.37 | 0.7084 |
| **0** | 1 (inferior-anterior) | 5 (superior-tubular) | 0.84 | 0.4038 |
| **0** | 2 (superior-anterior) | 3 (posterior) | 0.70 | 0.4840 |
| **0** | 2 (superior-anterior) | 4 (inferior-tubular) | -0.88 | 0.3798 |
| **0** | 2 (superior-anterior) | 5 (superior-tubular) | -0.42 | 0.6763 |
| **0** | 3 (posterior) | 4 (inferior-tubular) | -1.58 | 0.1147 |
| **0** | 3 (posterior) | 5 (superior-tubular) | -1.12 | 0.2639 |
| **0** | 4 (inferior-tubular) | 5 (superior-tubular) | 0.46 | 0.6449 |
| **0.16** | 1 (inferior-anterior) | 2 (superior-anterior) | -0.13 | 0.8947 |
| **0.16** | 1 (inferior-anterior) | 3 (posterior) | -1.20 | 0.2287 |
| **0.16** | 1 (inferior-anterior) | 4 (inferior-tubular) | 0.14 | 0.8910 |
| **0.16** | 1 (inferior-anterior) | 5 (superior-tubular) | -1.20 | 0.2305 |
| **0.16** | 2 (superior-anterior) | 3 (posterior) | -1.07 | 0.2839 |
| **0.16** | 2 (superior-anterior) | 4 (inferior-tubular) | 0.27 | 0.7877 |
| **0.16** | 2 (superior-anterior) | 5 (superior-tubular) | -1.07 | 0.2860 |
| **0.16** | 3 (posterior) | 4 (inferior-tubular) | 1.34 | 0.1801 |
| **0.16** | 3 (posterior) | 5 (superior-tubular) | 0.00 | 0.9964 |
| **0.16** | 4 (inferior-tubular) | 5 (superior-tubular) | -1.34 | 0.1816 |
| **37** | 1 (inferior-anterior) | 2 (superior-anterior) | -0.29 | 0.7715 |
| **37** | 1 (inferior-anterior) | 3 (posterior) | -1.25 | 0.2105 |
| **37** | 1 (inferior-anterior) | 4 (inferior-tubular) | 0.05 | 0.9621 |
| **37** | 1 (inferior-anterior) | 5 (superior-tubular) | -0.64 | 0.5225 |
| **37** | 2 (superior-anterior) | 3 (posterior) | -0.96 | 0.3366 |
| **37** | 2 (superior-anterior) | 4 (inferior-tubular) | 0.34 | 0.7346 |
| **37** | 2 (superior-anterior) | 5 (superior-tubular) | -0.35 | 0.7279 |
| **37** | 3 (posterior) | 4 (inferior-tubular) | 1.31 | 0.1919 |
| **37** | 3 (posterior) | 5 (superior-tubular) | 0.62 | 0.5382 |
| **37** | 4 (inferior-tubular) | 5 (superior-tubular) | -0.69 | 0.4904 |
| **92** | 1 (inferior-anterior) | 2 (superior-anterior) | 1.01 | 0.3107 |
| **92** | 1 (inferior-anterior) | 3 (posterior) | -1.24 | 0.2161 |
| **92** | 1 (inferior-anterior) | 4 (inferior-tubular) | 1.34 | 0.1801 |
| **92** | 1 (inferior-anterior) | 5 (superior-tubular) | 0.17 | 0.8668 |
| **92** | 2 (superior-anterior) | 3 (posterior) | -2.25 | **0.0246** |
| **92** | 2 (superior-anterior) | 4 (inferior-tubular) | 0.33 | 0.7438 |
| **92** | 2 (superior-anterior) | 5 (superior-tubular) | -0.85 | 0.3975 |
| **92** | 3 (posterior) | 4 (inferior-tubular) | 2.58 | **0.0101** |
| **92** | 3 (posterior) | 5 (superior-tubular) | 1.41 | 0.1602 |
| **92** | 4 (inferior-tubular) | 5 (superior-tubular) | -1.17 | 0.2409 |
| **190** | 1 (inferior-anterior) | 2 (superior-anterior) | -0.56 | 0.5782 |
| **190** | 1 (inferior-anterior) | 3 (posterior) | -2.80 | **0.0053** |
| **190** | 1 (inferior-anterior) | 4 (inferior-tubular) | 0.35 | 0.7229 |
| **190** | 1 (inferior-anterior) | 5 (superior-tubular) | -1.45 | 0.1479 |
| **190** | 2 (superior-anterior) | 3 (posterior) | -2.24 | **0.0254** |
| **190** | 2 (superior-anterior) | 4 (inferior-tubular) | 0.91 | 0.3626 |
| **190** | 2 (superior-anterior) | 5 (superior-tubular) | -0.89 | 0.3727 |
| **190** | 3 (posterior) | 4 (inferior-tubular) | 3.15 | **0.0017** |
| **190** | 3 (posterior) | 5 (superior-tubular) | 1.35 | 0.1781 |
| **190** | 4 (inferior-tubular) | 5 (superior-tubular) | -1.80 | 0.0718 |

**Supplementary Table S1f. Post hoc contrasts between hypothalamus subpart for each illuminance during the emotional task**

| **Illuminance** | **subpart** | **vs. subpart** | **t-value** | **p-value** |
| --- | --- | --- | --- | --- |
| **0** | 1 (inferior-anterior) | 2 (superior-anterior) | 0.45 | 0.6504 |
| **0** | 1 (inferior-anterior) | 3 (posterior) | 0.56 | 0.5775 |
| **0** | 1 (inferior-anterior) | 4 (inferior-tubular) | -0.34 | 0.7355 |
| **0** | 1 (inferior-anterior) | 5 (superior-tubular) | -1.50 | 0.1349 |
| **0** | 2 (superior-anterior) | 3 (posterior) | 0.10 | 0.9173 |
| **0** | 2 (superior-anterior) | 4 (inferior-tubular) | -0.79 | 0.4289 |
| **0** | 2 (superior-anterior) | 5 (superior-tubular) | -1.95 | 0.0515 |
| **0** | 3 (posterior) | 4 (inferior-tubular) | -0.90 | 0.3709 |
| **0** | 3 (posterior) | 5 (superior-tubular) | -2.05 | **0.0403** |
| **0** | 4 (inferior-tubular) | 5 (superior-tubular) | -1.16 | 0.2470 |
| **0.16** | 1 (inferior-anterior) | 2 (superior-anterior) | 1.38 | 0.1684 |
| **0.16** | 1 (inferior-anterior) | 3 (posterior) | 0.52 | 0.6049 |
| **0.16** | 1 (inferior-anterior) | 4 (inferior-tubular) | 0.76 | 0.4455 |
| **0.16** | 1 (inferior-anterior) | 5 (superior-tubular) | -0.42 | 0.6773 |
| **0.16** | 2 (superior-anterior) | 3 (posterior) | -0.86 | 0.3896 |
| **0.16** | 2 (superior-anterior) | 4 (inferior-tubular) | -0.62 | 0.5387 |
| **0.16** | 2 (superior-anterior) | 5 (superior-tubular) | -1.79 | 0.0730 |
| **0.16** | 3 (posterior) | 4 (inferior-tubular) | 0.25 | 0.8059 |
| **0.16** | 3 (posterior) | 5 (superior-tubular) | -0.93 | 0.3507 |
| **0.16** | 4 (inferior-tubular) | 5 (superior-tubular) | -1.18 | 0.2385 |
| **37** | 1 (inferior-anterior) | 2 (superior-anterior) | 0.32 | 0.7454 |
| **37** | 1 (inferior-anterior) | 3 (posterior) | -0.45 | 0.6497 |
| **37** | 1 (inferior-anterior) | 4 (inferior-tubular) | -0.58 | 0.5602 |
| **37** | 1 (inferior-anterior) | 5 (superior-tubular) | -1.01 | 0.3136 |
| **37** | 2 (superior-anterior) | 3 (posterior) | -0.78 | 0.4361 |
| **37** | 2 (superior-anterior) | 4 (inferior-tubular) | -0.91 | 0.3644 |
| **37** | 2 (superior-anterior) | 5 (superior-tubular) | -1.33 | 0.1828 |
| **37** | 3 (posterior) | 4 (inferior-tubular) | -0.13 | 0.8979 |
| **37** | 3 (posterior) | 5 (superior-tubular) | -0.55 | 0.5798 |
| **37** | 4 (inferior-tubular) | 5 (superior-tubular) | -0.43 | 0.6706 |
| **92** | 1 (inferior-anterior) | 2 (superior-anterior) | 1.19 | 0.2355 |
| **92** | 1 (inferior-anterior) | 3 (posterior) | -1.35 | 0.1788 |
| **92** | 1 (inferior-anterior) | 4 (inferior-tubular) | 0.11 | 0.9111 |
| **92** | 1 (inferior-anterior) | 5 (superior-tubular) | -1.77 | 0.0772 |
| **92** | 2 (superior-anterior) | 3 (posterior) | -2.53 | **0.0115** |
| **92** | 2 (superior-anterior) | 4 (inferior-tubular) | -1.08 | 0.2825 |
| **92** | 2 (superior-anterior) | 5 (superior-tubular) | -2.96 | **0.0032** |
| **92** | 3 (posterior) | 4 (inferior-tubular) | 1.46 | 0.1454 |
| **92** | 3 (posterior) | 5 (superior-tubular) | -0.42 | 0.6721 |
| **92** | 4 (inferior-tubular) | 5 (superior-tubular) | -1.88 | 0.0603 |
| **190** | 1 (inferior-anterior) | 2 (superior-anterior) | 1.54 | 0.1237 |
| **190** | 1 (inferior-anterior) | 3 (posterior) | -3.31 | **0.0010** |
| **190** | 1 (inferior-anterior) | 4 (inferior-tubular) | -0.18 | 0.8549 |
| **190** | 1 (inferior-anterior) | 5 (superior-tubular) | -2.50 | **0.0126** |
| **190** | 2 (superior-anterior) | 3 (posterior) | -4.85 | **<.0001** |
| **190** | 2 (superior-anterior) | 4 (inferior-tubular) | -1.72 | 0.0851 |
| **190** | 2 (superior-anterior) | 5 (superior-tubular) | -4.04 | **<.0001** |
| **190** | 3 (posterior) | 4 (inferior-tubular) | 3.13 | **0.0018** |
| **190** | 3 (posterior) | 5 (superior-tubular) | 0.81 | 0.4182 |
| **190** | 4 (inferior-tubular) | 5 (superior-tubular) | -2.32 | **0.0208** |

**Supplementary Table S1g. Association between performance to the 2-back task and the activity of each hypothalamus subpart during each illuminance**

|  | **F-value** | **p-value** | **Partial R²** |
| --- | --- | --- | --- |
| **1 (inferior-anterior hypothalamus subpart)** | | | |
| **Subpart activity** | < 0.01 | 0.99 |  |
| **Illuminance** | 1.94 | 0.13 |  |
| **Age** | 0.04 | 0.84 |  |
| **Sex** | 6.43 | **0.019** | 0.23 |
| **BMI** | 2.02 | 0.16 |  |
| **2 (superior-anterior hypothalamus subpart)** | | | |
| **Subpart activity** | 0.62 | 0.43 |  |
| **Illuminance** | 2.24 | 0.07 |  |
| **Age** | 0.01 | 0.94 |  |
| **Sex** | 6.36 | **0.019** | 0.22 |
| **BMI** | 2.04 | 0.17 |  |
| **3 (Posterior hypothalamus subpart)** | | | |
| **Subpart activity** | 9.43 | **0.0027** | 0.08 |
| **Illuminance** | 2.72 | **0.034** | 0.1 |
| **Age** | 0.04 | 0.85 |  |
| **Sex** | 6.07 | **0.022** | 0.21 |
| **BMI** | 1.82 | 0.19 |  |
| **4 (inferior-tubular hypothalamus subpart)** | | | |
| **Subpart activity** | 0.12 | 0.7 |  |
| **Illuminance** | 2.09 | 0.11 |  |
| **Age** | 0.03 | 0.86 |  |
| **Sex** | 6.54 | **0.018** | 0.23 |
| **BMI** | 2.01 | 0.17 |  |
| **5 (superior-tubular hypothalamus subpart)** | | | |
| **Subpart activity** | 0.25 | 0.62 |  |
| **Illuminance** | 2.12 | 0.084 |  |
| **Age** | 0.02 | 0.88 |  |
| **Sex** | 6.1 | **0.021** | 0.21 |
| **BMI** | 2.01 | 0.17 |  |
